# Glyphosate Promotes the Spread of Antibiotic Resistance Genes in the Intestine: An Overlooked Environmental Risk

**DOI:** 10.3390/toxics14060506

**Published:** 2026-06-10

**Authors:** Junyue Zheng, Xiangguang Chen, Jiazhen Jiang, Fengchang Wu

**Affiliations:** 1 National-Regional Joint Engineering Research Center for Soil Pollution Control and Remediation in South China, Guangdong Key Laboratory of Integrated Agro-Environmental Pollution Control and Management, Institute of Eco-environmental and Soil Sciences, Guangdong Academy of Sciences, Guangzhou 510650, China; junyuezheng@126.com; 2 Innovation Center of Pesticide Research, Department of Applied Chemistry, College of Sciences, China Agricultural University, Beijing 100193, China; jiangjiazhen@cau.edu.cn

**Keywords:** glyphosate, oxytetracycline, antibiotic resistance genes, aminomethylphosphonic acid, combined toxicity, risk assessment

## Abstract

Glyphosate (Gly) is currently the most commonly used broad-spectrum herbicide in the world. The extensive residues of Gly and its major metabolite aminomethylphosphonic acid (AMPA) in food and the environment make it inevitable for humans to consume them. Although Gly has been shown to disturb the homeostasis of gut microbiome by inhibiting the shikimic acid pathway of microorganisms, the potential health effects [such as the occurrence of antibiotic resistance genes (ARGs)] remain unclear. Furthermore, as antibiotics that also act on the intestinal microbiota, their extensive residues inevitably lead to co-exposure with Gly. For these reasons, this study used zebrafish as experimental organisms to explore the effects of Gly/AMPA and oxytetracycline (OTC) exposure alone or in combination on ARGs in the intestine. Our results indicate that Gly exposure, rather than AMPA exposure, led to a 1.67-fold increase in the relative abundance of ARGs in the zebrafish intestine. Combined exposure to Gly and OTC led to a 2.30-fold increase in the relative abundance of ARGs in the zebrafish intestine, indicating a synergistic effect, whereas the additive effect of AMPA and OTC was negligible. In conclusion, the health risk of antibiotic resistance caused by Gly through the gut microbiota is a neglected hot topic. Further studies are needed to clarify Gly-induced functional drug resistance and to assess the human relevance of these findings using more appropriate model organisms.

## 1. Introduction

As the most commonly used broad-spectrum herbicide globally, glyphosate (Gly) degrades primarily to aminomethylphosphonic acid (AMPA) [1,2,3]. Its widespread use has resulted in widespread contamination, with both the Gly and AMPA detected in food [4,5], air [6], dust [6], and water [7]. Gly is present in a range of food products, with the highest levels typically found in grains [8] and legumes [9]. It is also detectable in various fruits [4], vegetables [10], and in breast-milk substitutes such as infant formula [11]. The presence of Gly in human urine confirms inevitable exposure in humans [12].

The main mechanism of Gly as a herbicide is the inhibition of 5-enolpyruvylshikimate-3-phosphate synthase in the shikimic acid pathway (SKAP) [13]. Since the SKAP does not exist in human and animal cells, Gly is considered to be highly safe. However, the SKAP exists in some microorganisms and is essential for their survival [14,15]. Studies have shown that exposure to Gly inhibits the shikimate pathway in intestinal microbiota and alters its community structure, for example, in honey bees [16], cows [17], pigs [18], and rats [19]. And Gly mainly accumulates in the intestines and is mainly degraded into AMPA [20,21]. Therefore, the adverse health effects of Gly and AMPA resulting from intestinal flora disruption deserve close attention.

Antibiotic resistance has captured global attention and is considered among the most urgent healthcare crises of our time [22,23,24]. Encoded by antibiotic resistance genes (ARGs), microbial antibiotic resistance can spread across different environments through the exchange of ARGs with pathogens via mobile genetic elements (MGEs) [25,26,27], ultimately increasing the likelihood of deadly epidemics. This threat is further aggravated by the widespread presence of antibiotics, along with other toxic environmental pollutants [28,29]. Research indicates that exposure to Gly enriches ARGs and MGEs in soil microbiota [30]. However, two key questions remain: the individual effects of Gly and AMPA on ARGs in the intestinal microbiota, and their combined effects with antibiotics.

Among numerous broad-spectrum antibacterial agents, oxytetracycline (OTC), due to its tetracycline antibiotic properties, has become one of the most common residues in food and the environment. According to reports, OTC concentrations in soil from southern China, surface water from southwestern China, and surface water from northern China are 287 mg/kg [31], 237.0 ng/L [32], and 361.1 μg/L [33], respectively. In addition, OTC is also frequently detected in food, including milk in India (196.6–206.9 μg/L) [34], eggs in Nigeria (421.0–568.0 μg/kg) [35], honey in Turkey (89.0–92.0 μg/kg) [36], and seafood in the United States (2.7–8.6 μg/kg) [37]. Given that antibiotics and Gly are frequently detected in the environment and food, and that they both act on intestinal microbiota, it is necessary to study the combined effect of Gly and antibiotics on ARGs in the intestinal microbiota.

In this study, zebrafish were used as the experimental subjects. Based on environmentally relevant concentrations—specifically, the average concentration of Gly in freshwater aquaculture ponds (10 μg/L) [38], the maximum concentration of AMPA in surface water of agricultural watersheds (10 μg/L) [39], and the average concentration of OTC in the Yangtze River and Taihu Lake (3.0 μg/L) [40]—we investigated the effects of single and combined exposure on ARGs in the intestinal microbiota. The exposures included Gly alone (10 μg/L), AMPA alone (10 μg/L), OTC alone (3 μg/L), as well as binary mixtures of Gly + OTC and AMPA + OTC.

## 2. Materials and Methods

### 2.1. Reagents

The study utilized glyphosate (CAS: 1071-83-6, 99.5%) and oxytetracycline (CAS: 79-57-2, ≥98%) sourced from Aladdin (Shanghai, China), together with AMPA (CAS: 1066-51-9, 98.0%) sourced from Macklin (Shanghai, China). Other chemicals were either analytical or chromatographic grade.

### 2.2. Experimental Design

Adult zebrafish (wild type, 4 months old) were obtained from Beijing Hongda Gaofeng Aquarium (Beijing, China). They were reared under controlled conditions: a 14 h:10 h light–dark photoperiod and a constant temperature of 27 ± 1 °C. The fish were fed twice daily. After one week of acclimation to these conditions, the exposure experiment began. A total of 720 zebrafish were randomly assigned to six experimental groups (three biological replicates per group, 40 fish per replicate): a control group, an OTC-exposure group, a Gly exposure group, a Gly + OTC co-exposure group, an AMPA-exposure group, and an AMPA + OTC co-exposure group. The exposure concentrations were set at 10.00 μg/L for both Gly and AMPA, and 3.00 μg/L for OTC. These levels correspond to the average concentration of Gly reported in freshwater aquaculture ponds [38], the maximum concentration of AMPA reported in surface water from agricultural watersheds [39], and the average concentration of OTC reported in the Yangtze River and Tai Lake [40], respectively. Fish from each replicate were exposed to 20 L of the respective exposure solution for 28 days. During the experiment, three-quarters of the exposure solution was replaced every 24 h. All six experimental groups received 0.0015% dimethyl sulfoxide (DMSO). The experiment was conducted according to the guidelines of the Animal Care Institution and Usage Committee of China Agricultural University and approved by the Independent Animal Ethics Committee of China Agricultural University (protocol code Aw80105202-6-05 and date of approval 8 January 2025).

### 2.3. Sample Collection and DNA Extraction

At the end of the experiment, zebrafish were anesthetized with 0.02% MS-222 (Sigma-Aldrich, St. Louis, MO, USA), immediately immersed in absolute ethanol for a few seconds, and then rinsed five times with sterile water to minimize microbial interference from the body wall. For the evaluation of ARG diversity and abundance in the zebrafish intestine, the intestinal samples of three fish from the same tank were combined as one biological replicate. Three biological replicates were set up for each experimental group. The remaining fish from this exposure experiment were used for the conduct of our other experiment [41]. Under sterile conditions, the fish were dissected on ice using sterile instruments to excise the intestines, which were then transferred into 2 mL Eppendorf tubes. All collected samples were flash-frozen in liquid nitrogen and stored at −80 °C until further analysis. Genomic DNA was extracted from the intestinal samples using the FastDNA Spin Kit for Soil (MP Biomedicals, Irvine, CA, USA) following the manufacturer’s instructions. The extracted DNA was preserved at −20 °C until use.

### 2.4. HT-qPCR of ARGs

To assess the diversity and abundance of ARGs in the zebrafish gut, HT-qPCR was performed using the WaferGen SmartChip real-time PCR system (Wafergen Inc., Fremont, CA, USA). The HT-qPCR program and the analysis of normalized copy number of ARGs and the fold-change value of ARGs (FC value) were carried out as described previously [42]. A total of 296 primer sets were included in each run, targeting 285 ARGs, 10 mobile genetic elements (MGEs), and the 16S rRNA gene (Table S1). For each run (each plate), a non-template control was amplified in triplicate for every primer pair. For each biological replicate, there are three technical replicates in each plate. The reaction mixture consisted of nuclease-free PCR-grade water, 1 × LightCycler 480 SYBR Green I Master mix, bovine serum albumin, primers, and DNA template. The thermal cycling protocol followed a previously described method [43], starting with an initial denaturation at 95 °C for 10 min, followed by 40 cycles of 95 °C for 30 s and 60 °C for 30 s for annealing. Melting curve analysis and qPCR data were processed using the instrument’s software (SmartChip qPCR Software Version 2.8.6.1, San Jose, CA, USA). Only wells showing amplification efficiencies between 90% and 110% were considered valid. If only one out of three technical replicates failed to amplify, the corresponding ARG was excluded from further analysis. The enrichment of ARGs in treated guts relative to the control was expressed as the fold-change (FC) value, as described in a previous study [44]. A threshold cycle (CT) value (31) was set as the detection limit, and the relative copy number of ARGs and FC value were calculated according to the formula.

R = relative gene copy number = 10^((31-CT)/(10/3))^

Normalized ARG copy number = (R/relative l6S rRNA gene copy number) × 4.1

ΔCT= CT_(ARG)_ − CT_(16S)_

ΔΔCT= CT_(target)_ − ΔCT_(ref)_

FC = 2^(−ΔΔCT)^

CT is the threshold cycle, target is the amended sample, and ref is the control sample [44]. To minimize potential variation in DNA extraction efficiencies, the relative abundance of ARGs was normalized by the 16S rRNA gene and converted to ARG copies per cell, of which 4.1 is the average number of 16S rRNA gene copies per bacterium [45]. Detailed steps for HT-qPCR of ARGs are provided in the Supplementary Information. Based on the 16S RNA sequencing data we have published [41], a network analysis was conducted on the co-occurrence patterns among ARG subtypes, MGEs, and bacterial taxa.

### 2.5. Statistical Analysis

Statistical analyses were conducted using SPSS (version 26.0, IBM, Armonk, NY, USA). A one-way ANOVA followed by Fisher and Duncan’s post hoc tests was applied, with statistical significance set at *p* < 0.05. Additionally, the Bliss independence model was employed to determine the types of interaction present in the mixture groups [46].

(1) $$P_{OTC}=\frac{\left|OTC-Control\right|\times 100\%}{Control}$$

(2) $$P_{Gly}=\frac{\left|Gly-Control\right|\times 100\%}{Control}$$

(3) $$P_{AMPA}=\frac{\left|AMPA-Control\right|\times 100\%}{Control}$$

(4) $$P_{MIX}=P_{OTC}+P_{Gly}-P_{OTC}\times P_{Gly}\;\mathrm{or}\;P_{MIX}=P_{OTC}+P_{AMPA}-P_{OTC}\times P_{AMPA}$$

The individual fractional effects of OTC, Gly, and AMPA—denoted as *P_OTC_*, *P_Gly_*, and *P_AMPA_*, respectively—were determined relative to the solvent control (expressed as % increase or decrease). The predicted combined fractional effect for binary mixtures (OTC + Gly or OTC + AMPA), termed *P_MIX_*, was calculated accordingly. The experimentally measured combined effect (*P_DME_*) was then compared with *P_MIX_*. *P_DME_* > *P_MIX_* indicates that the combined effect of Gly and OTC is greater than the sum of their individual effects. Therefore, there is a synergistic effect (SYN) between Gly and OTC. *P_DME_* < *P_MIX_* indicates that the combined effect of Gly and OTC is less than the sum of their individual effects. Gly interferes with or inhibits the action of OTC, and there is an antagonistic effect (ANT) between Gly and OTC. *P_DME_* ≈ *P_MIX_*, which indicates that the mechanisms of action of Gly and OTC are the same. Their combined effect is the total effect of dose addition. Gly and OTC have an additive effect (AE) on each other [47].

## 3. Results

### 3.1. Diversity, Abundance, and Enrichment of ARGs

**Figure 1:**
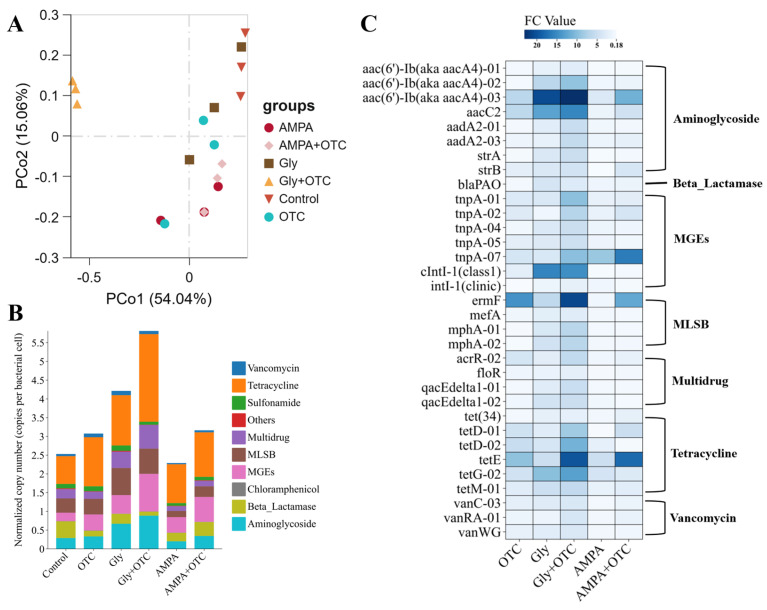
(**A**) PCoA based on the Bray–Curtis distance showing the distribution pattern of ARGs in the intestines. (**B**) The normalized copy number of ARGs in the intestines. (**C**) Heat maps of ARGs enrichment in the treated intestines compared to the control. FC value was used to show the significant decrease or enrichment of ARGs. A result of 2^(−ΔΔCt)^ > 1 was considered enrichment. A result of 2^(−ΔΔCt)^ < 1 was considered a decrease.

According to Figure 1A, PCoA based on Bray–Curtis distance revealed clear separations of ARG profiles in the zebrafish gut among groups treated with OTC, Gly, Gly + OTC, AMPA, and AMPA + OTC from the control. To minimize experimental deviation, normalized copy numbers of ARGs and MGEs per bacterial cell were used to represent their abundances. As shown in Figure 1B, total ARG levels in the Gly and Gly + OTC groups were approximately ~1.67- and 2.30-fold higher than the control, respectively. Specifically, as shown in Table 1, tetracycline resistance gene abundances increased to ~1.76-, 1.81-, and 3.15-fold of the control in the OTC, Gly, and Gly + OTC groups, respectively. MGE abundances increased to ~1.90-, 2.20-, 4.47-, 1.84-, and 2.96-fold; multidrug resistance gene abundances increased only in the Gly and Gly + OTC groups, to ~1.75- and 2.53-fold; and aminoglycoside resistance gene abundances increased in the same two groups, to ~2.33- and 3.07-fold of the control, respectively.

We used the absolute value of the mean fold change in ARGs to verify their enrichment (Figure 1C). In the contaminant-exposed zebrafish intestines, the relative abundance of tetracycline resistance genes (tetE and tetG-02) was markedly higher than in the control. Among these, tetE showed the most pronounced increase, reaching 19.89-fold (Gly + OTC) and 18.05-fold (AMPA + OTC) relative to the control. Aminoglycoside resistance genes (aac(6′)-Ib-03 and aacC2) were significantly enriched in the Gly alone and Gly + OTC groups. Notably, the Gly + OTC treatment resulted in a distinct ARG enrichment profile that differed from both Gly alone and OTC alone.

According to Table 2, the combination of Gly and OTC exerted synergistic effects on the abundances of aminoglycoside, multidrug, sulfonamide, tetracycline, chloramphenicol, MGEs, and total ARGs. In contrast, Table 3 shows that while AMPA and OTC acted synergistically on MGE abundance, they displayed antagonistic effects on the abundances of aminoglycoside, multidrug, sulfonamide, tetracycline, vancomycin, and total ARGs.

### 3.2. Relationship Patterns Between ARGs, MGEs, and Bacterial Taxa

**Figure 2:**
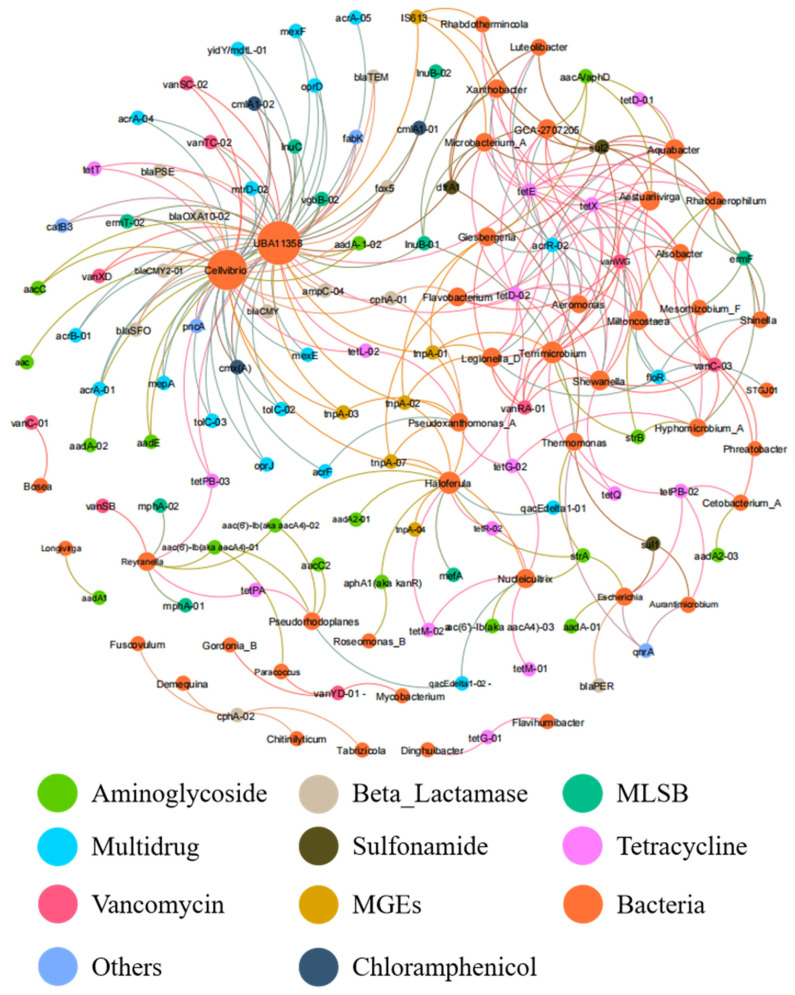
Network analysis for the co-occurrence patterns between ARG subtypes, MGEs, and bacterial taxa at the genus level. A connection represents a strong (Spearman’s correlation coefficient r  >  0.5) and significant (*p*  <  0.05) correlation. Nodes represent ARGs, MGEs, and bacterial taxa; the size of each node is proportional to its number of connections.

According to Table 4, Spearman’s correlation analysis revealed a strong positive association between MGE abundance and total ARG abundance. A similar significant correlation was also observed between MGEs and several ARG categories (aminoglycoside, MLSB, multidrug, and tetracycline resistance genes) in the zebrafish gut. Additionally, the modular network structure at the genus level (Figure 2) linked ARGs to several bacterial taxa—*UBA11358*, *Cellvibrio*, *Xanthobacter*, *Miltoncostaea*, and *Aquabacter*—which were closely associated with multiple ARGs.

## 4. Discussion

Our previous research has shown that both Gly and AMPA can alter the composition of the gut microbiome in zebrafish, and this interfering effect is more significant when they are respectively exposed in combination with OTC [41]. This study further reveals that exposure to Gly and OTC increased the relative abundance of ARGs in the zebrafish intestine, in contrast to AMPA, which shows minimal effect. Firstly, exposure to tetracycline antibiotic OTC alone significantly increased the abundance of tetracycline ARGs in the intestine, which is in line with earlier research that demonstrated that antibiotics can cause the appearance of ARGs even at low antibiotic concentrations and spread [43,48]. Secondly, exposure to Gly alone significantly increased the diversity and relative abundance of ARGs in the intestine, which is similar to the previously reported Gly increase in the abundance of ARGs in soil [30]. One possible mechanism underlying the elevated relative abundance of ARGs is increased membrane permeability. Previous reports indicate that herbicides can increase bacterial cell membrane permeability, thereby facilitating plasmid movement [49]. This may explain the enhanced spread of tetracycline resistance genes (e.g., tetE) in the combined Gly and OTC exposure groups. In this study, the relative abundance of MGEs in the intestine of the combined treatment of Gly and antibiotics was much higher than that in other treatments. Through horizontal gene transfer, ARGs can be passed from bacteria that produce antibiotics to non-resistant microbes, and the major contributor to horizontal gene transfer is MGEs [50,51]. Alternatively, Gly may exert selective pressure on intestinal microorganisms by affecting the SKAP. Previous studies have shown that combined exposure to pollutants and antibiotics exerts co-selective pressure on the gut microbiota, ultimately leading to an increased relative abundance of ARGs [42]. In this study, combined exposure to Gly and OTC revealed a statistically significant association between the intestinal bacterial community and ARGs. This association, however, represents co-occurrence rather than causation. Subsequent functional studies are warranted to explore the causal links between these associated bacteria and ARGs.

Due to the fact that Gly and OTC are frequently found in food and water [4,8,9,40], ingestion through contaminated food and drinking water is considered to be the main route of human exposure to Gly and OTC. For example, the Gly content in wheat and barley in Denmark was detected to be 1.62 mg/kg and 1.25 mg/kg, respectively [8]. Residues of Gly in soy infant formula from Brazil ranged from 0.03 mg/kg to 1.08 mg/kg, and residues of AMPA ranged from 0.02 mg/kg to 0.17 mg/kg [11]. In addition, the detection of Gly in the urine of occupationally exposed persons (0.26–76 µg/L) and environmentally exposed persons (0.16–7.6 µg/L) also indicates that humans are at risk of long-term exposure to Gly [52]. Similarly, the levels of OTC in seafood in the US market range from 2.7 to 8.6 μg/kg, and the estimated daily intake of OTC through consumption of contaminated seafood ranges from 0.141 to 0.447 μg/d per capita [37]. Given the inevitable human exposure to Gly and OTC via food and water, this study demonstrates that these compounds increase the relative abundance of ARGs in the zebrafish intestine. Nevertheless, further mammalian studies are warranted to evaluate the implications for human health risk.

## 5. Conclusions

Our results show that Gly, but not its metabolite AMPA, significantly increased the relative abundance of ARGs in the zebrafish intestine. Furthermore, Gly and OTC acted synergistically in this effect. Gly and antibiotics are widely detected in the environment and food, leading to their inevitable ingestion by humans. As these findings are based on zebrafish, further mammalian studies are needed to evaluate human exposure risk.

## Figures and Tables

**Table 1:** The normalized copy number of ARGs in the gut after 28 days exposure to control, OTC, Gly, Gly + OTC, AMPA, and AMPA + OTC, respectively (mean ± SD, *n* = 3).

Group	Aminoglycoside	Multidrug	Beta_Lactamase	Others	Sulfonamide
Control	0.29 ± 0.15 ^b^	0.25 ± 0.12 ^b^	0.45 ± 0.17 ^a^	0.02 ± 0.01 ^a^	0.12 ± 0.06 ^a^
OTC	0.33 ± 0.18 ^b^	0.19 ± 0.10 ^b^	0.15 ± 0.09 ^bc^	0.01 ± 0.01 ^a^	0.13 ± 0.09 ^a^
Gly	0.67 ± 0.16 ^a^	0.44 ± 0.04 ^ab^	0.26 ± 0.07 ^abc^	0.02 ± 0.01 ^a^	0.14 ± 0.03 ^a^
Gly + OTC	0.89 ± 0.18 ^a^	0.63 ± 0.31 ^a^	0.11 ± 0.03 ^c^	0.01 ± 0.00 ^a^	0.08 ± 0.02 ^a^
AMPA	0.20 ± 0.05 ^b^	0.13 ± 0.02 ^b^	0.23 ± 0.10 ^abc^	0.01 ± 0.00 ^a^	0.07 ± 0.02 ^a^
AMPA + OTC	0.34 ± 0.08 ^b^	0.15 ± 0.01 ^b^	0.37 ± 0.13 ^ab^	0.01 ± 0.00 ^a^	0.09 ± 0.01 ^a^
	MLSB	Tetracycline	MEGs	Vancomycin	Chloramphenicol
Control	0.38 ± 0.15 ^c^	0.74 ± 0.10 ^b^	0.22 ± 0.10 ^c^	0.06 ± 0.02 ^a^	0.01 ± 0.00 ^a^
OTC	0.42 ± 0.22 ^bc^	1.31 ± 0.42 ^b^	0.43 ± 0.18 ^bc^	0.10 ± 0.06 ^a^	0.01 ± 0.01 ^a^
Gly	0.72 ± 0.08 ^a^	1.34 ± 0.13 ^b^	0.49 ± 0.09 ^bc^	0.11 ± 0.06 ^a^	0.01 ± 0.00 ^a^
Gly + OTC	0.67 ± 0.08 ^ab^	2.34 ± 0.48 ^a^	1.00 ± 0.12 ^a^	0.08 ± 0.03 ^a^	0.00 ± 0.00 ^a^
AMPA	0.17 ± 0.04 ^c^	1.04 ± 0.2 ^b^	0.41 ± 0.06 ^bc^	0.03 ± 0.01 ^a^	0.01 ± 0.00 ^a^
AMPA + OTC	0.29 ± 0.10 ^c^	1.19 ± 0.14 ^b^	0.66 ± 0.15 ^b^	0.05 ± 0.01 ^a^	0.01 ± 0.00 ^a^

**Note:** In the same indicator, if two treatment groups contain the same superscript letter, it indicates insignificance (*p* ≥ 0.05); otherwise, it indicates significance (*p* < 0.05).

**Table 2:** Types of interactions between OTC and Gly at different endpoints.

Endpoints	*P* ^a^		*P_MIX_*	*P_DME_*	Mixture Effects
	OTC	Gly			
Aminoglycoside	0.14	1.31	1.27	2.07	SYN
Multidrug	0.24	0.76	0.82	1.52	SYN
Beta_Lactamase	0.67	0.42	0.81	0.76	ANT
Others	0.50	0	0.50	0.50	AE
Sulfonamide	0.08	0.17	0.24	0.33	SYN
MLSB	0.10	0.89	0.90	0.76	ANT
Tetracycline	0.77	0.81	0.96	2.16	SYN
MEGs	0.95	1.23	1.01	3.55	SYN
Vancomycin	0.67	0.83	0.94	0.33	ANT
Chloramphenicol	0	0	0	1	SYN
Total ARGs	0.15	0.61	0.67	1.08	SYN

**^a^** The observed fractional effect of OTC or Gly alone.

**Table 3:** Types of interactions between OTC and AMPA at different endpoints.

Endpoints	*P* ^a^		*P_MIX_*	*P_DME_*	Mixture Effects
	OTC	AMPA			
Aminoglycoside	0.14	0.31	0.40	0.17	ANT
Multidrug	0.24	0.48	0.60	0.40	ANT
Beta_Lactamase	0.67	0.49	0.83	0.18	ANT
Others	0.50	0.50	0.75	0.50	ANT
Sulfonamide	0.08	0.42	0.47	0.25	ANT
MLSB	0.10	0.55	0.60	0.27	ANT
Tetracycline	0.77	0.40	0.86	0.61	ANT
MEGs	0.95	0.86	0.99	2.0	SYN
Vancomycin	0.67	0.50	0.84	0.17	ANT
Chloramphenicol	0	0	0	0	AE
Total ARGs	0.15	0.19	0.31	0.08	ANT

**^a^** The observed fractional effect of OTC or AMPA alone.

**Table 4:** Spearman’s correlation examining the association between MGE and ARG relative abundances in zebrafish intestines.

	MGEs	
	*R*-Value	*p*-Value
Aminoglycoside	0.69	<0.05
Beta_Lactamase	−0.232	0.354
Chloramphenicol	−0.212	0.399
MLSB	0.488	<0.05
Multidrug	0.521	<0.05
Others	−0.224	0.372
Sulfonamide	−0.015	0.951
Tetracycline	0.789	<0.001
Vancomycin	0.267	0.284
Total ARGs	0.738	<0.001

## Data Availability

The original contributions presented in this study are included in the article/Supplementary Material. Further inquiries can be directed to the corresponding authors.

## Supplementary Materials

The following supporting information can be downloaded at: https://www.mdpi.com/article/10.3390/toxics14060506/s1, Table S1: A list of primers used to amplify ARGs and MGEs in this study.
