# Supplementary material for: Glyphosate Promotes the Spread of Antibiotic Resistance Genes in the Intestine: An Overlooked Environmental Risk
Source: Toxics. 2026 Jun 10;14(6):506. doi: 10.3390/toxics14060506 (PMC13307782; doi:10.3390/toxics14060506)
Supplement: Supplementary file 1 [file toxics-14-00506-s001.zip › toxics-4317231-supplementary.pdf]

Supporting Information:

Glyphosate Promotes the Spread of Antibiotic Resistance Genes in the Intestine: An Overlooked Environmental Risk

Junyue Zheng [1], Xiangguang Chen [1,*], Jiazhen Jiang [2] and Fengchang Wu [1,*]

[1] National-Regional Joint Engineering Research Center for Soil Pollution Control and Remediation in South China, Guangdong Key Laboratory of Integrated Agro-Environmental Pollution Control and Management, Institute of Eco-environmental and Soil Sciences, Guangdong Academy of Sciences, Guangzhou 510650, China; junyuezheng@126.com

[2] Innovation Center of Pesticide Research, Department of Applied Chemistry, College of Sciences, China Agricultural University, Beijing 100193, China; jiangjiazhen@cau.edu.cn

* Correspondence: chenxiangguang@126.com (X.C.); wufengchang@vip.skleg.cn (F.W.)

Summary of Supporting Information

Table

Table S1. A list of primers used to amplify ARGs and MGEs in this study.

TABLE

Table S1. A list of primers used to amplify ARGs and MGEs in this study.

No.	Gene Name	Forward Primer	Reverse Primer	Classification
1	16S rRNA	GGGTTGCGCTCGTTGC	ATGGYGTCGTCAGCTCGTG	16S rRNA
2	aac	CCCTGCGTTGTGGCTATGT	TTGGCCACGCCAATCC	Aminoglycoside
3	aac(6')I1	GACCGGATTAAGGCCGATG	CTTGCCTTGATATTCAGTTTTTATAACCA	Aminoglycoside
4	aac(6')-Ib(aka aacA4)-01	GTTTGAGAGGCAAGGTACCGTAA	GAATGCCTGGCGTGTTTGA	Aminoglycoside
5	aac(6')-Ib(aka aacA4)-02	CGTCGCCGAGCAACTTG	CGGTACCTTGCCTCTCAAACC	Aminoglycoside
6	aac(6')-Ib(aka aacA4)-03	AGAAGCACGCCCGACACTT	GCTCTCCATTCAGCATTGCA	Aminoglycoside
7	aac(6')-II	CGACCCGACTCCGAACAA	GCACGAATCCTGCCTTCTCA	Aminoglycoside
8	aac(6')-Iy	GCTTTGCGGATGCCTCAAT	GGAGAACAAAAATACCTTCAAGGAAA	Aminoglycoside
9	aacA/aphD	AGAGCCTTGGGAAGATGAAGTTT	TTGATCCATACCATAGACTATCTCATCA	Aminoglycoside
10	aacC	CGTCACTTATTCGATGCCCTTAC	GTCGGGCGCGGCATA	Aminoglycoside
11	aacC1	GGTCGTGAGTTCGGAGACGTA	GCAAGTTCCCGAGGTAATCG	Aminoglycoside
12	aacC2	ACGGCATTCTCGATTGCTTT	CCGAGCTTCACGTAAGCATTT	Aminoglycoside
13	aacC4	CGGCGTGGGACACGAT	AGGGAACCTTTGCCATCAACT	Aminoglycoside
14	aadA-01	GTTGTGCACGACGACATCATT	GGCTCGAAGATACCTGCAAGAA	Aminoglycoside
15	aadA-02	CGAGATTCTCCGCGCTGTA	GCTGCCATTCTCCAAATTGC	Aminoglycoside
16	aadA1	AGCTAAGCGCGAACTGCAAT	TGGCTCGAAGATACCTGCAA	Aminoglycoside
17	aadA-1-01	AAAAGCCCGAAGAGGAACTTG	CATCTTTCACAAAGATGTTGCTGTCT	Aminoglycoside
18	aadA-1-02	CGGAATTGAAAAAACTGATCGAA	ATACCGGCTGTCCGTCATTT	Aminoglycoside
19	aadA2-01	ACGGCTCCGCAGTGGAT	GGCCACAGTAACCAACAAATCA	Aminoglycoside
20	aadA2-02	CTTGTCGTGCATGACGACATC	TCGAAGATACCCGCAAGAATG	Aminoglycoside
21	aadA2-03	CAATGACATTCTTGCGGGTATC	GACCTACCAAGGCAACGCTATG	Aminoglycoside
22	aadA5-01	ATCACGATCTTGCGATTTTGCT	CTGCGGATGGGCCTAGAAG	Aminoglycoside
23	aadA5-02	GTTCTTGCTCTTGCTCGCATT	GATGCTCGGCAGGCAAAC	Aminoglycoside
24	aadA9-01	CGCGGCAAGCCTATCTTG	CAAATCAGCGACCGCAGACT	Aminoglycoside
25	aadA9-02	GGATGCACGCTTGGATGAA	CCTCTAGCGGCCGGAGTATT	Aminoglycoside
26	aadD	CCGACAACATTTCTACCATCCTT	ACCGAAGCGCTCGTCGTATA	Aminoglycoside
27	aadE	TACCTTATTGCCCTTGGAAGAGTTA	GGAACTATGTCCCTTTTAATTCTACAATCT	Aminoglycoside
28	acrA-01	CAACGATCGGACGGGTTTC	TGGCGATGCCACCGTACT	Multidrug
29	acrA-02	GGTCTATCACCCTACGCGCTATC	GCGCGCACGAACATACC	Multidrug
30	acrA-03	CAGACCCGCATCGCATATT	CGACAATTTCGCGCTCATG	Multidrug
31	acrA-04	TACTTTGCGCGCCATCTTC	CGTGCGCGAACGAACAT	Multidrug
32	acrA-05	CGTGCGCGAACGAACA	ACTTTGCGCGCCATCTTC	Multidrug
33	acrB-01	AGTCGGTGTTCGCCGTTAAC	CAAGGAAACGAACGCAATACC	Multidrug
34	acrF	GCGGCCAGGCACAAAA	TACGCTCTTCCCACGGTTTC	Multidrug
35	acrR-01	GCGCTGGAGACACGACAAC	GCCTTGCTGCGAGAACAAA	Multidrug
36	acrR-02	GATGATACCCCCTGCTGTGAGA	ACCAAACAAGAAGCGCAAGAA	Multidrug
37	adeA	CAGTTCGAGCGCCTATTTCTG	CGCCCTGACCGACCAAT	Multidrug
38	ampC/blaDHA	TGGCCGCAGCAGAAAGA	CCGTTTTATGCACCCAGGAA	Beta_Lactamase
39	ampC-01	TGGCGTATCGGGTCAATGT	CTCCACGGGCCAGTTGAG	Beta_Lactamase
40	ampC-02	GCAGCACGCCCCGTAA	TGTACCCATGATGCGCGTACT	Beta_Lactamase
41	ampC-04	TCCGGTGACGCGACAGA	CAGCACGCCGGTGAAAGT	Beta_Lactamase
42	ampC-05	CTGTTCGAGCTGGGTTCTATAAGTAAA	CAGTATCTGGTCACCGGATCGT	Beta_Lactamase

43	ampC-06	CCGCTCAAGCTGGACCATAC	CCATATCCTGCACGTTGGTTT	Beta_Lactamase
44	ampC-07	CCGCCCAGAGCAAGGACTA	GCTCGACTTCACGCCGTAAG	Beta_Lactamase
45	ampC-09	CAGCCGCTGATGAAAAAATATG	CAGCGAGCCCACTTCGA	Beta_Lactamase
46	aph	TTTCAGCAAGTGGATCATGTTAAAAT	CCAAGCTGTTTCCACTGTTTTTC	Aminoglycoside
47	aph(2')-Id-01	TGAGCAGTATCATAAGTTGAGTGAAAAG	GACAGAACAATCAATCTCTATGGAATG	Aminoglycoside
48	aph(2')-Id-02	TAAGGATATACCGACAGTTTTGGAAA	TTTAATCCCTCTTCATACCAATCCATA	Aminoglycoside
49	aph6ia	CCCATCCCATGTGTAAGGAAA	GCCACCGCTTCTGCTGTAC	Aminoglycoside
50	aphA1(aka kanR)	TGAACAAGTCTGGAAAGAAATGCA	CCTATTAATTTCCCCTCGTCAAAAA	Aminoglycoside
51	bacA-01	CGGCTTCGTGACCTCGTT	ACAATGCGATACCAGGCAAAT	Others
52	bacA-02	TTCCACGACACGATTAAGTCATTG	CGGCTCTTTCGGCTTCAG	Others
53	bla1	GCAAGTTGAAGCGAAAGAAAAGA	TACCAGTATCAATCGCATATACACCTAA	Beta_Lactamase
54	bla-ACC-1	CACACAGCTGATGGCTTATCTAAAA	AATAAACGCGATGGGTTCCA	Beta_Lactamase
55	blaCMY	CCGCGGCGAAATTAAGC	GCCACTGTTTGCCTGTCAGTT	Beta_Lactamase
56	blaCMY2-01	AAAGCCTCAT GGGTGCATAAA	ATAGCTTTTGTTTGCCAGCATCA	Beta_Lactamase
57	blaCMY2-02	GCGAGCAGCCTGAAGCA	CGGATGGGCTTGTCCTCTT	Beta_Lactamase
58	blaCTX-M-01	GGAGGCGTGACGGCTTTT	TTCAGTGCGATCCAGACGAA	Beta_Lactamase
59	blaCTX-M-02	GCCGCGGTGCTGAAGA	ATCGGATTATAGTTAACCAGGTCAGATTT	Beta_Lactamase
60	blaCTX-M-03	CGATACCACCACGCCGTTA	GCATTGCCCAACGTCAGATT	Beta_Lactamase
61	blaCTX-M-04	CTTGGCGTTGCGCTGAT	CGTTCATCGGCACGGTAGA	Beta_Lactamase
62	blaCTX-M-05	GCGATAACGTGGCGATGAAT	GTCGAGACGGAACGTTTCGT	Beta_Lactamase
63	blaCTX-M-06	CACAGTTGGTGACGTGGCTTAA	CTCCGCTGCCGGTTTTATC	Beta_Lactamase
64	blaGES	GCAATGTGCTCAACGTTCAAG	GTGCCTGAGTCAATTCTTTCAAAG	Beta_Lactamase
65	blaIMP-01	AACACGGTTTGGTGGTTCTTGTA	GCGCTCCACAAACCAATTG	Beta_Lactamase
66	blaIMP-02	AAGGCAGCATTTCCTCTCATTTT	GGATAGATCGAGAATTAAGCCACTCT	Beta_Lactamase
67	bla-L1	CACCGGGTTACCAGCTGAAG	GCGAAGCTGCGCTTGTAGTC	Beta_Lactamase
68	blaMOX/blaCMY	CTATGTCAATGTGCCGAAGCA	GGCTTGTCCTCTTTCGAATAGC	Beta_Lactamase
69	blaOCH	GGCGACTTGCGCCGTAT	TTTTCTGCTCGGCCATGAG	Beta_Lactamase
70	blaOKP	GCCGCCATCACCATGAG	GGTGACGTTGTCACCGATCTG	Beta_Lactamase
71	blaOXA1/blaOXA30	CGGATGGTTTGAAGGGTTTATTAT	TCTTGGCTTTTATGCTTGATGTTAA	Beta_Lactamase
72	blaOXA10-01	CGCAATTATCGGCCTAGAAACT	TTGGCTTTCCGTCCCATTT	Beta_Lactamase
73	blaOXA10-02	CGCAATTATCGGCCTAGAAACT	TTGGCTTTCCGTCCCATTT	Beta_Lactamase
74	blaOXY	CGTTCAGGCGGCAGGTT	GCCGCGATATAAGATTTGAGAATT	Beta_Lactamase
75	blaPAO	CGCCCGTACAACCGGTGAT	GAAGTAATGCGGTTCTCCTTTCA	Beta_Lactamase
76	blaPER	TGCTGGTTGCTGTTTTTGTGA	CCTGCGCAATGATAGCTTCAT	Beta_Lactamase
77	blaPSE	TTGTGACCTATTCCCCTGTAATAGAA	TGCGAAGCACGCATCATC	Beta_Lactamase
78	blaROB	GCAAAGGCATGACGATTGC	CGCGCTGTTGTCGCTAAA	Beta_Lactamase
79	blaSFO	CCGCCGCCATCCAGTA	GGGCCGCCAAGATGCT	Beta_Lactamase
80	blaSHV-01	TCCCATGATGAGCACCTTTAAA	TTCGTCACCGGCATCCA	Beta_Lactamase
81	blaSHV-02	CTTTCCCATGATGAGCACCTTT	TCCTGCTGGCGATAGTGGAT	Beta_Lactamase
82	blaTEM	AGCATCTTACGGATGGCATGA	TCCTCCGATCGTTGTCAGAAGT	Beta_Lactamase
83	blaTLA	ACACTTTGCCATTGCTGTTTATGT	TGCAAATTTCGGCAATAATCTTT	Beta_Lactamase
84	blaVEB	CCCGATGCAAAGCGTTATG	GAAAGATTCCCTTTATCTATCTCAGACAA	Beta_Lactamase
85	blaVIM	GCACTTCTCGCGGAGATTG	CGACGGTGATGCGTACGTT	Beta_Lactamase

86	blaZ	GGAGATAAAGTAACAAATCCAGTTAGATATGA	TGCTTAATTTTCCATTTGCGATAAG	Beta_Lactamase
87	carB	GGAGTGAGGCTGACCGTAGAAG	TGCTTAATTTTCCATTTGCGATAAG	MLSB
88	catA1	GGGTGAGTTTCACCAGTTTTGATT	CACCTTGTCGCCTTGCGTATA	Others
89	catB3	GCACTCGATGCCTTCCAAAA	AGAGCCGATCCAAACGTCAT	Others
90	catB8	CACTCGACGCCTTCCAAAG	CCGAGCCTATCCAGACATCATT	Others
91	ceoA	ATCAACACGGACCAGGACAAG	GGAAAGTCCGCTCACGATGA	Multidrug
92	cepA	AGTTGCGCAGAACAGTCCTCTT	TCGTATCTTGCCCGTCGATAAT	Beta_Lactamase
93	cfiA	GCAGCGTTGCTGGACACA	GTTCGGGATAAACGTGGTGACT	Beta_Lactamase
94	cfr	GCAAAATTCAGAGCAAGTTACGAA	AAAATGACTCCCAACCTGCTTTAT	Others
95	cfxA	TCATTCCTCGTTCAAGTTTTCAGA	TGCAGCACCAAGAGGAGATGT	Beta_Lactamase
96	intI-1(clinic)	GGCATCCAAGCAGCAAG	AAGCAGACTTGACCTGA	MGEs
97	cmeA	GCAGCAAAGAAGAAGCACCAA	AGCAGGGTAAGTAAAACTAAGTGGTAAATCT	Multidrug
98	cmlA1-01	TAGGAAGCATCGGAACGTTGAT	CAGACCGAGCACGACTGTTG	Chloramphenicol
99	cmlA1-02	AGGAAGCATCGGAACGTTGA	ACAGACCGAGCACGACTGTTG	Chloramphenicol
100	cmr	CGGCATCGTCAGTGGAATT	CGGTTCCGAAAAAGATGGAA	Multidrug
101	cmx(A)	GCGATCGCCATCCTCTGT	TCGACACGGAGCCTTGGT	Chloramphenicol
102	cphA-01	GCGAGCTGCACAAGCTGAT	CGGCCCAGTCGCTCTTC	Beta_Lactamase
103	cphA-02	GTGCTGATGGCGAGTTTCTG	GGTGTGGTAGTTGGTGTTGATCAC	Beta_Lactamase
104	dfrA1	GGAATGGCCCTGATATTCCA	AGTCTTGCGTCCAACCAACAG	Sulfonamide
105	dfrA12	CCTCTACCGAACCGTCACACA	GCGACAGCGTTGAAACAACTAC	Sulfonamide
106	emrD	CTCAGCAGTATGGTGGTAAGCATT	ACCAGGCGCCGAAGAAC	Multidrug
107	ereA	CCTGTGGTACGGAGAATTCATGT	ACCGCATTCGCTTTGCTT	MLSB
108	ereB	GCTTTATTTCAGGAGGCGGAAT	TTTTAAATGCCACAGCACAGAATC	Others
109	erm(34)	GCGCGTTGACGACGATTT	TGGTCATACTCGACGGCTAGAAC	MLSB
110	erm(35)	TTGAAAACGATGTTGCATTAAGTCA	TCTATAATCACAACTAACCACTTGAACGT	MLSB
111	erm(36)	GGCGGACCGACTTGCAT	TCTGCGTTGACGACGGTTAC	MLSB
112	ermA	TTGAGAAGGGATTTGCGAAAAG	ATATCCATCTCCACCATTAATAGTAAACC	MLSB
113	ermA/ermTR	ACATTTTACCAAGGAACTTGTGGAA	GTGGCATGACATAAACCTTCATCA	MLSB
114	ermB	TAAAGGGCATTTAACGACGAAACT	TTTATACCTCTGTTTGTTAGGGAATTGAA	MLSB
115	ermC	TTTGAAATCGGCTCAGGAAAA	ATGGTCTATTTCAATGGCAGTTACG	MLSB
116	ermF	CAGCTTTGGTTGAACATTTACGAA	AAATTCCTAAAATCACAACCGACAA	MLSB
117	ermJ/ermD	GGACTCGGCAATGGTCAGAA	CCCCGAAACGCAATATAATGTT	MLSB
118	ermK-01	GTTTGATATTGGCATTGTCAGAGAAA	ACCATTGCCGAGTCCACTTT	MLSB
119	ermK-02	GAGCCGCAAGCCCCTTT	GTGTTTCATTTGACGCGGAGTAA	MLSB
120	ermT-01	GTTCACTAGCACTATTTTTAATGACAGAAGT	GAAGGGTGTCTTTTTAATACAATTAACGA	MLSB
121	ermT-02	GTAAAATCCCTAGAGAATACTTTCATCCA	TGAGTGATATTTTTGAAGGGTGTCTT	MLSB
122	ermX	GCTCAGTGGTCCCCATGGT	ATCCCCCCGTCAACGTTT	MLSB
123	ermY	TTGTCTTTGAAAGTGAAGCAACAGT	TAACGCTAGAGAACGATTTGTATTGAG	MLSB
124	fabK	TTTCAGCTCAGCACTTTGGTCAT	AAGGCATCTTTTTCAGCCAGTTC	Others
125	floR	ATTGTCTTCACGGTGTCCGTTA	CCGCGATGTCGTCGAACT	Multidrug
126	folA	CGAGCAGTTCCTGCCAAAG	CCCAGTCATCCGGTTCATAATC	Sulfonamide
127	fosB	TCACTGTAACTAATGAAGCATTAGACCAT	CCATCTGGATCTGTAAAGTAAAGAGATC	Others
128	fosX	GATTAAGCCATATCACTTTAATTGTGAAAG	TCTCCTTCCATAATGCAAATCCA	Others

129	fox5	GGTTTGCCGCTGCAGTTC	GCGGCCAGGTGACCAA	Beta_Lactamase
130	imiR	CCGGACTAGAGCTTCATGTAAGC	CCCACGCGGTACTCTTGTAAA	Others
131	cIntI-1(class1)	CGAACGAGTGGCGGAGGGTG	TACCCGAGAGCTTGGCACCCA	MGEs
132	IS613	AGGTTCGGACTCAATGCAACA	TTCAGCACATACCGCCTTGAT	MGEs
133	lmrA-01	TCGACGTGACCGTAGTGAACA	CGTGACTACCCAGGTGAGTTGA	MLSB
134	lnuA-01	TGACGCTCAACACACTCAAAAA	TTCATGCTTAAGTTCCATACGTGAA	MLSB
135	lnuB-01	TGAACATAATCCCCTCGTTTAAAGAT	TAATTGCCCTGTTTCATCGTAAATAA	MLSB
136	lnuB-02	AAAGGAGAAGGTGACCAATACTCTGA	GGAGCTACGTCAAACAACCAGTT	MLSB
137	lnuC	TGGTCAATATAACAGATGTAAACCAGATTT	CACCCCAGCCACCATCAA	MLSB
138	marR-01	GCGGCGTACTGGTGAAGCTA	TGCCCTGGTCGTTGATGA	Multidrug
139	matA/mel	TAGTAGGCAAGCTCGGTGTTGA	CCTGTGCTATTTTAAGCCTTGTTTCT	MLSB
140	mdetl1	ATACAGCAGTGGATATTGGTTTAATTGT	TGCATAAGGTGAATGTTCCATGA	Multidrug
141	mdtA	CCTAACGGGCGTGACTTCA	TTCACCTGTTTCAAGGGTCAAA	MLSB
142	mdtE/yhiU	CGTCGGCGCACTCGTT	TCCAGACGTTGTACGGTAACCA	Multidrug
143	mecA	GGTTACGGACAAGGTGAAATACTGAT	TGTCTTTTAATAAGTGAGGTGCGTTAATA	Beta_Lactamase
144	mefA	CCGTAGCATTGGAACAGCTTTT	AAACGGAGTATAAGAGTGCTGCAA	MLSB
145	mepA	ATCGGTCGCTCTTCGTTCAC	ATAAATAGGATCGAGCTGCTGGAT	Multidrug
146	mexA	AGGACAACGCTATGCAACGAA	CCGGAAAGGGCCGAAAT	Multidrug
147	mexD	TTGCCACTGGCTTTCATGAG	CACTGCGGAGAACTGTCTGTAGA	Multidrug
148	mexE	GGTCAGCACCGACAAGGTCTAC	AGCTCGACGTACTTGAGGAACAC	Multidrug
149	mexF	CCGCGAGAAGGCCAAGA	TTGAGTTCGGCGGTGATGA	Multidrug
150	mphA-01	CTGACGCGCTCCGTGTT	GGTGGTGCATGGCGATCT	MLSB
151	mphA-02	TGATGACCCTGCCATCGA	TTCGCGAGCCCCTCTTC	MLSB
152	mphB	CGCAGCGCTTGATCTTGTAG	TTACTGCATCCATACGCTGCTT	MLSB
153	mphC	CGTTTGAAGTACCGAATTGGAAA	GCTGCGGGTTTGCCTGTA	MLSB
154	msrA-01	CTGCTAACACAAGTACGATTCCAAAT	TCAAGTAAAGTTGTCTTACCTACACCATT	MLSB
155	msrC-01	TCAGACCGGATCGGTTGTC	CCTATTTTTTGGAGTCTTCTCTCTAATGTT	MLSB
156	mtrC-01	GGACGGGAAGATGGTCCAA	CGTAGCGTTCCGGTTCGAT	Multidrug
157	mtrC-02	CGGAGTCCATCGACCATTTG	ATCGTCGGCAAGGAGAATCA	Multidrug
158	mtrD-02	GGTCGGCACGCTCTTGTC	TGAAGAATTTGCGCACCACTAC	Multidrug
159	mtrD-03	CCGCCAAGCCGATATAGACA	GGCCGGGTTGCCAAA	Multidrug
160	ndm-1	ATTAGCCGCTGCATTGAT	CATGTCGAGATAGGAAGTG	Beta_Lactamase
161	nimE	TGCGCCAAGATAGGGCATA	GTCGTGAATTCGGCAGGTTTA	Others
162	nisB	GGGAGAGTTGCCGATGTTGTA	AGCCACTCGTTAAAGGGCAAT	Others
163	oleC	CCCGGAGTCGATGTTCGA	GCCGAAGACGTACACGAACAG	MLSB
164	oprD	ATGAAGTGGAGCGCCATTG	GGCCACGGCGAACTGA	Multidrug
165	oprJ	ACGAGAGTGGCGTCGACAA	AAGGCGATCTCGTTGAGGAA	Multidrug
166	pbp	CCGGTGCCATTGGTTTAGA	AAAATAGCCGCCCCAAGATT	Beta_Lactamase
167	pbp2x	TTTCATAAGTATCTGGACATGGAAGAA	CCAAAGGAAACTTGCTTGAGATTAG	Beta_Lactamase
168	Pbp5	GGCGAACTTCTAATTAATCCTATCCA	CGCCGATGACATTCTTCTTATCTT	Beta_Lactamase
169	penA	AGACGGTAACGTATAACTTTTTGAAAGA	GCGTGTAGCCGGCAATG	Beta_Lactamase
170	pikR1	TCGACATGCGTGACGAGATT	CCGCGAATTAGGCCAGAA	MLSB
171	pikR2	TCGTGGGCCAGGTGAAGA	TTCCCCTTGCCGGTGAA	MLSB

172	pmrA	TTTGCAGGTTTTGTTCCTAATGC	GCAGAGCCTGATTTCTCCTTTG	Multidrug
173	pncA	GCAATCGAGGCGGTGTTC	TTGCCGCAGCCAATTCA	Others
174	putitive multidrug	AATTTTGCCGATTATTGCTGAAA	GATTGTCATCATTCGTTTATCACCAA	Multidrug
175	qac	CAATAATAACCGAAATAATAGGGACAAGTT	AATAAGTGTTCCTAGTGTTGGCCATAG	Multidrug
176	qacA	TGGCAATAGGAGCTATGGTGTTT	AAGGTAACACTATTTTCGGTCCAAATC	Multidrug
177	qacA/qacB	TTTAGGCAGCCTCGCTTCA	CCGAATCCAAATAAAACCCAATAA	Multidrug
178	qacEdelta1-01	TCGCAACATCCGCATTAAAA	ATGGATTTCAGAACCAGAGAAAGAAA	Multidrug
179	qacEdelta1-02	CCCCTTCCGCCGTTGT	CGACCAGACTGCATAAGCAACA	Multidrug
180	qacH-01	GTGGCAGCTATCGCTTGGAT	CCAACGAACGCCCACAA	Multidrug
181	qacH-02	CATCGTGCTTGTGGCAGCTA	TGAACGCCCAGAAGTCTAGTTTT	Multidrug
182	qnrA	AGGATTTCTCACGCCAGGATT	CCGCTTTCAATGAAACTGCAA	Others
183	rarD-02	TGACGCATCGCGTGATCT	AAATTTTCTGTGGCGTCTGAATC	Multidrug
184	sat4	GAATGGGCAAAGCATAAAAACTTG	CCGATTTTGAAACCACAATTATGATA	Others
185	sdeB	CACTACCGCTTCCGCACTTAA	TGAAAAAAACGGGAAAAGTCCAT	Multidrug
186	spcN-01	AAAAGTTCGATGAAACACGCCTAT	TCCAGTGGTAGTCCCCGAATC	Aminoglycoside
187	spcN-02	CAGAATCTTCCTGAAAAGTTTGATGAA	CGCAGACACGCCGAATC	Aminoglycoside
188	speA	GCAAGAGGTATTTGCTCAACAAGA	CAGGGTCACCCTCATAAAGAAAA	Others
189	str	AATGAGTTTTGGAGTGTCTCAACGTA	AATCAAAACCCCTATTAAAGCCAAT	Aminoglycoside
190	strA	CCGGTGGCATTTGAGAAAAA	GTGGCTCAACCTGCGAAAAG	Aminoglycoside
191	strB	GCTCGGTCGTGAGAACAATCT	CAATTTCGGTCGCCTGGTAGT	Aminoglycoside
192	sul1	CAGCGCTATGCGCTCAAG	ATCCCGCTGCGCTGAGT	Sulfonamide
193	sul2	TCATCTGCCAAACTCGTCGTTA	GTCAAAGAACGCCGCAATGT	Sulfonamide
194	sulA/folP-01	CAGGCTCGTAAATTGATAGCAGAAG	CTTTCCTTGCGAATCGCTTT	Sulfonamide
195	sulA/folP-03	CACGGCTTCGGCTCATGT	TGCCATCCTGTGACTAGCTACGT	Sulfonamide
196	tet(32)	CCATTACTTCGGACAACGGTAGA	CAATCTCTGTGAGGGCATTTAACA	Tetracycline
197	tet(34)	CTTAGCGCAAACAGCAATCAGT	CGGTGATACAGCGCGTAAACT	Tetracycline
198	tet(35)	ACCCCATGACGTACCTGTAGAGA	CAACCCACACTGGCTACCAGTT	Tetracycline
199	tet(36)-01	AGAATACTCAGCAGAGGTCAGTTCCT	TGGTAGGTCGATAACCCGAAAAT	Tetracycline
200	tet(36)-02	TGCAGGAAAGACCTCCATTACAG	CTTTGTCCACACTTCCACGTACTATG	Tetracycline
201	tet(37)	GAGAACGTTGAAAAGGTGGTGAA	AACCAAGCCTGGATCAGTCTCA	Tetracycline
202	tetA-01	GCTGTTTGTTCTGCCGGAAA	GGTTAAGTTCCTTGAACGCAAACT	Tetracycline
203	tetA-02	CTCACCAGCCTGACCTCGAT	CACGTTGTTATAGAAGCCGCATAG	Tetracycline
204	tetB-01	AGTGCGCTTTGGATGCTGTA	AGCCCCAGTAGCTCCTGTGA	Tetracycline
205	tetB-02	GCCCAGTGCTGTTGTTGTCAT	TGAAAGCAAACGGCCTAAATACA	Tetracycline
206	tetC-01	CATATCGCAATACATGCGAAAAA	AAAGCCGCGGTAAATAGCAA	Tetracycline
207	tetC-02	ACTGGTAAGGTAAACGCCATTGTC	ATGCATAAACCAGCCATTGAGTAAG	Tetracycline
208	tetD-01	TGCCGCGTTTGATTACACA	CACCAGTGATCCCGGAGATAA	Tetracycline
209	tetD-02	TGTCATCGCGCTGGTGATT	CATCCGCTTCCGGGAGAT	Tetracycline
210	tetE	TTGGCGCTGTATGCAATGAT	CGACGACCTATGCGATCTGA	Tetracycline
211	tetG-01	TCAACCATTGCCGATTCGA	TGGCCCGGCAATCATG	Tetracycline
212	tetG-02	CATCAGCGCCGGTCTTATG	CCCCATGTAGCCGAACCA	Tetracycline
213	tetH	TTTGGGTCATCTTACCAGCATTAA	TTGCGCATTATCATCGACAGA	Tetracycline
214	tetJ	GGGTGCCGCATTAGATTACCT	TCGTCCAATGTAGAGCATCCATA	Tetracycline

215	tetK	CAGCAGTCATTGGAAAATTATCTGATTATA	CCTTGTACTAACCTACCAAAAATCAAAATA	Tetracycline
216	tetL-01	AGCCCGATTTATTCAAGGAATTG	CAAATGCTTTCCCCCTGTTCT	Tetracycline
217	tetL-02	ATGGTTGTAGTTGCGCGCTATAT	ATCGCTGGACCGACTCCTT	Tetracycline
218	tetM-01	CATCATAGACACGCCAGGACATAT	CGCCATCTTTTGCAGAAATCA	Tetracycline
219	tetM-02	TAATATTGGAGTTTTAGCTCATGTTGATG	CCTCTCTGACGTTCTAAAAGCGTATTAT	Tetracycline
220	tetO-01	ATGTGGATACTACAACGCATGAGATT	TGCCTCCACATGATATTTTTCCT	Tetracycline
221	tetPA	AGTTGCAGATGTGTATAGTCGTAAACTATCTATT	TGCTACAAGTACGAAAACAAAACTAGAA	Tetracycline
222	tetPB-01	ACACCTGGACACGCTGATTTT	ACCGTCTAGAACGCGGAATG	Tetracycline
223	tetPB-02	TGATACACCTGGACACGCTGAT	CGTCCAAAACGCGGAATG	Tetracycline
224	tetPB-03	TGGGCGACAGTAGGCTTAGAA	TGACCCTACTGAAACATTAGAAATATACCT	Tetracycline
225	tetPB-04	AGTGGTGCAAATACTGAAAAAGTTGT	TTTGTTCCTTCGTTTTGGACAGA	Tetracycline
226	tetPB-05	CTGAAGTGGAGCGATCATTCC	CCCTCAACGGCAGAAATAACTAA	Tetracycline
227	tetQ	CGCCTCAGAAGTAAGTTCATACACTAAG	TCGTTCATGCGGATATTATCAGAAT	Tetracycline
228	tetR-02	CGCGATAGACGCCTTCGA	TCCTGACAACGAGCCTCCTT	Tetracycline
229	tetR-03	CGCGATGGAGCAAAAGTACAT	AGTGAAAAACCTTGTTGGCATAAAA	Tetracycline
230	tetS	TTAAGGACAAACTTTCTGACGACATC	TGTCTCCCATTGTTCTGGTTCA	Tetracycline
231	tetT	CCATATAGAGGTTCCACCAAATCC	TGACCCTATTGGTAGTGGTTCTATTG	Tetracycline
232	tetU-01	GTGGCAAAGCAACGGATTG	TGCGGGCTTGCAAAACTATC	Tetracycline
233	tetV	GCGGGAACGACGATGTATATC	CCGCTATCTCACGACCATGAT	Tetracycline
234	tetX	AAATTTGTTACCGACACGGAAGTT	CATAGCTGAAAAAATCCAGGACAGTT	Tetracycline
235	tnpA-01	CATCATCGGACGGACAGAATT	GTCGGAGATGTGGGTGTAGAAAGT	MGEs
236	tnpA-02	GGGCGGGTCGATTGAAA	GTGGGCGGGATCTGCTT	MGEs
237	tnpA-03	AATTGATGCGGACGGCTTAA	TCACCAAACTGTTTATGGAGTCGTT	MGEs
238	tnpA-04	CCGATCACGGAAAGCTCAAG	GGCTCGCATGACTTCGAATC	MGEs
239	tnpA-05	GCCGCACTGTCGATTTTTATC	GCGGGATCTGCCACTTCTT	MGEs
240	tnpA-07	GAAACCGATGCTACAATATCCAATTT	CAGCACCGTTTGCAGTGTAAG	MGEs
241	tolC-01	GGCCGAGAACCTGATGCA	AGACTTACGCAATTCCGGGTTA	Multidrug
242	tolC-02	CAGGCAGAGAACCTGATGCA	CGCAATTCCGGGTTGCT	Multidrug
243	tolC-03	GCCAGGCAGAGAACCTGATG	CGCAATTCCGGGTTGCT	Multidrug
244	Tp614	GGAAATCAACGGCATCCAGTT	CATCCATGCGCTTTTGTCTCT	MGEs
245	ttgA	ACGCCAATGCCAAACGATT	GTCACGGCGCAGCTTGA	Multidrug
246	ttgB	TCGCCCTGGATGTACACCTT	ACCATTGCCGACATCAACAAC	Multidrug
247	vanA	AAAAGGCTCTGAAAACGCAGTTAT	CGGCCGTTATCTTGTAAAAACAT	Vancomycin
248	vanB-01	TTGTCGGCGAAGTGGATCA	AGCCTTTTTCCGGCTCGTT	Vancomycin
249	vanB-02	CCGGTCGAGGAACGAAATC	TCCTCCTGCAAAAAAAGATCAAC	Vancomycin
250	vanC-01	ACAGGGATTGGCTATGAACCAT	TGACTGGCGATGATTTGACTATG	Vancomycin
251	vanC-03	AAATCAATACTATGCCGGGCTTT	CCGACCGCTGCCATCA	Vancomycin
252	vanC1	AGGCGATAGCGGGTATTGAA	CAATCGTCAATTGCTCATTTCC	Vancomycin
253	vanC2/vanC3	TTTGACTGTCGGTGCTTGTGA	TCAATCGTTTCAGGCAATGG	Vancomycin
254	vanG	ATTTGAATTGGCAGGTATACAGGTTA	TGATTTGTCTTTGTCCATACATAATGC	Vancomycin
255	vanHB	GAGGTTTCCGAGGCGACAA	CTCTCGGCGGCAGTCGTAT	Vancomycin
256	vanHD	GTGGCCGATTATACCGTCATG	CGCAGGTCATTCAGGCAAT	Vancomycin
257	vanRA-01	CCCTTACTCCCACCGAGTTTT	TTCGTCGCCCCATATCTCAT	Vancomycin

258	vanRA-02	CCACTCCGGCCTTGTCATT	GCTAACCACATTCCCCTTGTTTT	Vancomycin
259	vanRB	GCCCTGTCGGATGACGAA	TTACATAGTCGTCTGCCTCTGCAT	Vancomycin
260	vanRC	TGCGGGAAAAACTGAACGA	CCCCCCATACGGTTTTGATTA	Vancomycin
261	vanRC4	AGTGCTTTGGCTTATCTCGAAAA	TCCGGCAGCATCACATCTAA	Vancomycin
262	vanRD	TTATAATGGCAAGGATGCACTAAAGT	CGTCTACATCCGGAAGCATGA	Vancomycin
263	vanSA	CGCGTCATGCTTTCAAAATTC	TCCGCAGAAAGCTCAATTTGTT	Vancomycin
264	vanSB	GCGCGGCAAATGACAAC	TTTGCCATTTTATTCGCACTGT	Vancomycin
265	vanSC-02	GCCATCAGCGAGTCTGATGA	CAGCTGGGATCGTTTTTCCTT	Vancomycin
266	vanSE	TGGCCGAAGAAGCAGGAA	CAATAATACTCGTCAAAGGAGTTCTCA	Vancomycin
267	vanTC-01	CACACGCATTTTTTCCCATCTAG	CAGCCAACAGATCATCAAAACAA	Vancomycin
268	vanTC-02	ACAGTTGCCGCTGGTGAAG	CGTGGCTGGTCGATCAAAA	Vancomycin
269	vanTE	GTGGTGCCAAGGAAGTTGCT	CGTAGCCACCGCAAAAAAAT	Vancomycin
270	vanTG	CGTGTAGCCGTTCCGTTCTT	CGGCATTACAGGTATATCTGGAAA	Vancomycin
271	vanWB	CGGACAAAGATACCCCCTATAAAG	AAATAGTAAATTGCTCATCTGGCACAT	Vancomycin
272	vanWG	ACATTTTCATTTTGGCAGCTTGTAC	CCGCCATAAGAGCCTACAATCT	Vancomycin
273	vanXA	CGCTAAATATGCCACTTGGGATA	TCAAAAGCGATTCAGCCAACT	Vancomycin
274	vanXB	AGGCACAAAATCGAAGATGCTT	GGGTATGGCTCATCAATCAACTT	Vancomycin
275	vanXD	TAAACCGTGTTATGGGAACGAA	GCGATAGCCGTCCCATAAGA	Vancomycin
276	vanYB	GGCTAAAGCGGAAGCAGAAA	GATATCCACAGCAAGACCAAGCT	Vancomycin
277	vanYD-01	AAGGCGATACCCTGACTGTCA	ATTGCCGGACGGAAGCA	Vancomycin
278	vanYD-02	CAAACGGAAGAGAGGTCACTTACA	CGGACGGTAATAGGGACTGTTC	Vancomycin
279	vatB-01	GGAAAAAGCAACTCCATCTCTTGA	TCCTGGCATAACAGTAACATTCTGA	MLSB
280	vatB-02	TTGGGAAAAAGCAACTCCATCT	CAATCCACACATCATTTCCAACA	MLSB
281	vatC-01	CGGAAATTGGGAACGATGTT	GCAATAATAGCCCCGTTTCCTA	MLSB
282	vatC-02	CGATGTTTGGATTGGACGAGAT	GCTGCAATAATAGCCCCGTTT	MLSB
283	vatE-01	GGTGCCATTATCGGAGCAAAT	TTGGATTGCCACCGACAAT	MLSB
284	vatE-02	GACCGTCCTACCAGGCGTAA	TTGGATTGCCACCGACAATT	MLSB
285	vgaA-01	CGAGTATTGTGGAAAGCAGCTAGTT	CCCGTACCGTTAGAGCCGATA	MLSB
286	vgaA-02	GACGGGTATTGTGGAAAGCAA	TTTCCTGTACCATTAGATCCGATAATT	MLSB
287	vgb-01	AGGGAGGGTATCCATGCAGAT	ACCAAATGCGCCCGTTT	MLSB
288	vgbB-01	CAGCCGGATTCTGGTCCTT	TACGATCTCCATTCAATTGGGTAAA	MLSB
289	vgbB-02	ATACGAGCTGCCTAATAAAGGATCTT	TGTGAACCACAGGGCATTATCA	MLSB
290	yceE/mdtG-01	TGGCACAAAATATCTGGCAGTT	TTGTGTGGCGATAAGAGCATTAG	Multidrug
291	yceE/mdtG-02	TTATCTGTTTTCTGCTCACCTTCTTTT	GCGTGGTGACAAACAGGCTTA	Multidrug
292	yceL/mdtH-01	TCGGGATGGTGGGCAAT	CGATAACCGAGCCGATGTAGA	Multidrug
293	yceL/mdtH-02	CGCGTGAAACCTTAAGTGCTT	AGACGGCTAAACCCCATATAGCT	Multidrug
294	yceL/mdtH-03	CTGCCGTTAAATGGATGTATGC	ACTCCAGCGGGCGATAGG	Multidrug
295	yidY/mdtL-01	GCAGTTGCATATCGCCTTCTC	CTTCCCGGCAAACAGCAT	Multidrug
296	yidY/mdtL-02	TGCTGATCGGGATTCTGATTG	CAGGCGCGACGAACATAAT	Multidrug
